# The Shelf Life of Skulls: Anthropology and ‘race’ in the Vrolik Craniological Collection

**DOI:** 10.1007/s10739-023-09716-w

**Published:** 2023-06-23

**Authors:** Laurens de Rooy

**Affiliations:** Museum Vrolik, Department of Medical Biology, Amsterdam University Medical Centers, Location AMC, Meibergdreef 15, 1105 AZ Amsterdam, The Netherlands

**Keywords:** Museum Vrolik, Skulls, Race, Blackness, Anthropology, Material culture

## Abstract

The Vrolik ethnographical collection consisted of roughly 300 skulls, mummified heads, skeletons, pelvises, wet-preserved preparations, and plaster models, collected by Gerard Vrolik (1775–1859) and his son Willem (1801–1863). Most prominent in this collection were the skulls, of which 177 remain in the collection of present-day Museum Vrolik. These skulls—a troubling heritage of colonialism and scientific racism—are the central subjects of this paper, which considers the changing meanings and values of these skulls for racial science over approximately 160 years, between ± 1800 and 1960. These shifting meanings are analysed using the skulls themselves as primary sources, including the labels, numbers and handwriting present on them or their stands. Central topics addressed will be matters of classification, hierarchy, scientific bias, and disciplinary development of racial anthropology from the study and collection of idealized national types to a quantitative craniometry of populations. This paper demonstrates that during 160 years of study of this same set of crania, the skulls of white European origin gradually lost racial relevance and were increasingly normalized, whereas the skulls of dark-skinned people of African descent continued to be categorized in a typological racial scheme and as such were increasingly othered.

## Introduction

*Museum Vrolikianum* was a large anatomical collection founded around 1800 by Amsterdam anatomist Gerard Vrolik (1775–1859) and expanded by his son Willem (1801–1863).^1^ By 1863, it contained more than 5000 skeletons, skulls, bones and anatomical preparations, including an so-called ethnographical collection of approximately 300 skulls, mummified heads, skeletons, pelvises, wet-preserved preparations and plaster models from across the world, classified by race. Skulls were most prominent in this collection; of these, 177 remain in the collection of the present-day Museum Vrolik.

The ethnographical collection has received interest from historians of race and anthropology (Hondius 2014, pp. 177–181; Schiebinger 1993, p. 158; Roque 2010, pp. 121 and 145; Sysling 2015a, pp. 27–31; Sysling 2015b). However, the collection has been difficult to study, as it was mostly catalogued only after Willem Vrolik’s death (Dusseau 1865).^2^ Although Willem finished the catalogue’s ethnographical portion (the *Partie Ethnographie*) during his lifetime, correspondence, notes and archival sources associated with the skulls are missing. Thus, elaborate object biographies or provenance studies of these remains are largely impossible. When donor names are mentioned, date or site of acquisition is seldom included.^3^

Given these limitations, this study interrogates the “shelf life”^4^ of Vrolik’s ethnographical collection through close study of the skulls themselves.^5^ Full object biographies are not the present focus, but rather the so-called lives of skulls once they enter the museum.^6^ Methodologically, such study entails systematic material analysis of the layered, iterative modifications of skulls as museum objects, including comparisons of handwriting, label styles and placement, preparation techniques, stand types, etc., alongside available published and archival documentary sources. Recently, Ricardo Roque has used a similar approach through his “micro-history of racial theory in a skull inscription” (Roque 2021, p. 728). Roque argues that the epistemological place of skull inscriptions—and, arguably, other physical markings on skulls—in racial skull collections “could surpass the pragmatics of museum storage, cataloguing, and even authentication; they constituted a deliberate writing practice that belongs to racial theory-making and classification” (Roque 2021, p. 727).

This paper’s aims to complement and elaborate Roque’s thesis: can study of the shelf life of skulls expose shifts in their meanings between the 1800’s and the 1950’s, as they were acquired and labelled, curated and studied, cleaned and sawn in half, relabeled and renumbered by anatomists, anthropologists and technicians? Can one trace changes in racial or typological categorization and scientific-racist hierarchy through changing uses of these remains as scientific objects? What changes in methodology are visible through shelf lives? For example, in the later nineteenth century, craniometry developed as a central method of physical anthropology, propelled by positivistic faith in the objectivity of quantitative approaches. In this paradigm, reliable results required many standardized measurements to calculate averages of large samples. Thus, collecting individual skulls became old-fashioned (Sysling 2015b, p. 197; Zimmerman 2001, pp. 87–88; Daston and Galison 2007).

Most directly, this study traces the shelf lives of the Vrolik skull collection. More broadly, this study attends to the development of European scientific racism. Skulls remained the same over time, but paradigms and practices framing their use, display, and modification were constantly changing. This, what I call close reading of skulls—as historical rather than osteological method—can contribute to a fuller understanding of scientific racism where documents are scarce. Shelf lives tell a story of changing meanings of race, understandings of the relationship between humans and apes, polygenism and monogenism, whiteness and blackness, supposed normal and other, and bias and supposed scientific objectivity.

This approach critically considers the practices and perspectives of the collector-scientists—all privileged, white Dutch scientists—who studied these racial skulls. Accounting for this troubling colonial heritage requires knowing in detail why these skulls were of value to the Vroliks and their successors, and what role they played in the rise of physical anthropology as a science of race.^7^

As such this study links to the existing literature on the history of (scientific) racism in relation to blackness and whiteness (e.g. Gould 1981; Blakey 1994; Curran 2011; Hondius 2014; Blakey 1994, 2020, 2021; Painter 2003, 2010; Mitchell 2018; Watkins 2018; Wekker 2020 etc.). The scientific construction of white supremacy can be traced back to an enlightenment notion of Eurocentric superiority, based on “scientific (and white) neutrality (..) [or] imagined absence of subjectivity” (Blakey 2021, p. 317), rooted in Christian religious convictions and in European aesthetic ideals (Blumenbach’s idealized Caucasian race (Painter 2003)). As Michael Blakey (2021, p. 317) argues the Christian justification of slavery and colonial oppression weakened around 1850 and was substituted by a scientific justification, based on the above mentioned supposedly objective and quantitative approaches of the emerging science of anthropology. At the same time the presumption of neutrality remained a constructed quality of whiteness (Frankenberg 1994). Unmarked and de-racialized, it equaled the privileged norm (Frankenberg 1994, 2001; Blakey 2021, p. 319; Wekker 2020, p. 354).

The following section attends to the various numbers and labels on the skulls and their significance. The next four sections follow approximately 150 years of the shelf life of these skulls, with each section centered around a main researcher or interval: Gerard Vrolik (ca. 1826), Willem Vrolik (1850–1860), Lodewijk Bolk (1900–1930), and the mid-twentieth century. Here, emphasis fixes on physical traces on the skulls themselves, in combination with available documentation. Through this method, the construction of racial classifications and hierarchies, and, particularly, the changing significance of skulls of white people (of European origin) in relation to those of Black people (of African descent) comes into focus.

## Reading Skulls

The Vrolik skull collection contains four different number types. The most fundamental is the so-called A-number, corresponding to the 1865 Dusseau catalogue. As previously mentioned, only this first catalogue section (*Partie Ethnographie*) was entirely written by Willem Vrolik. Willem only outlined the five other parts.

**Fig. 1:**
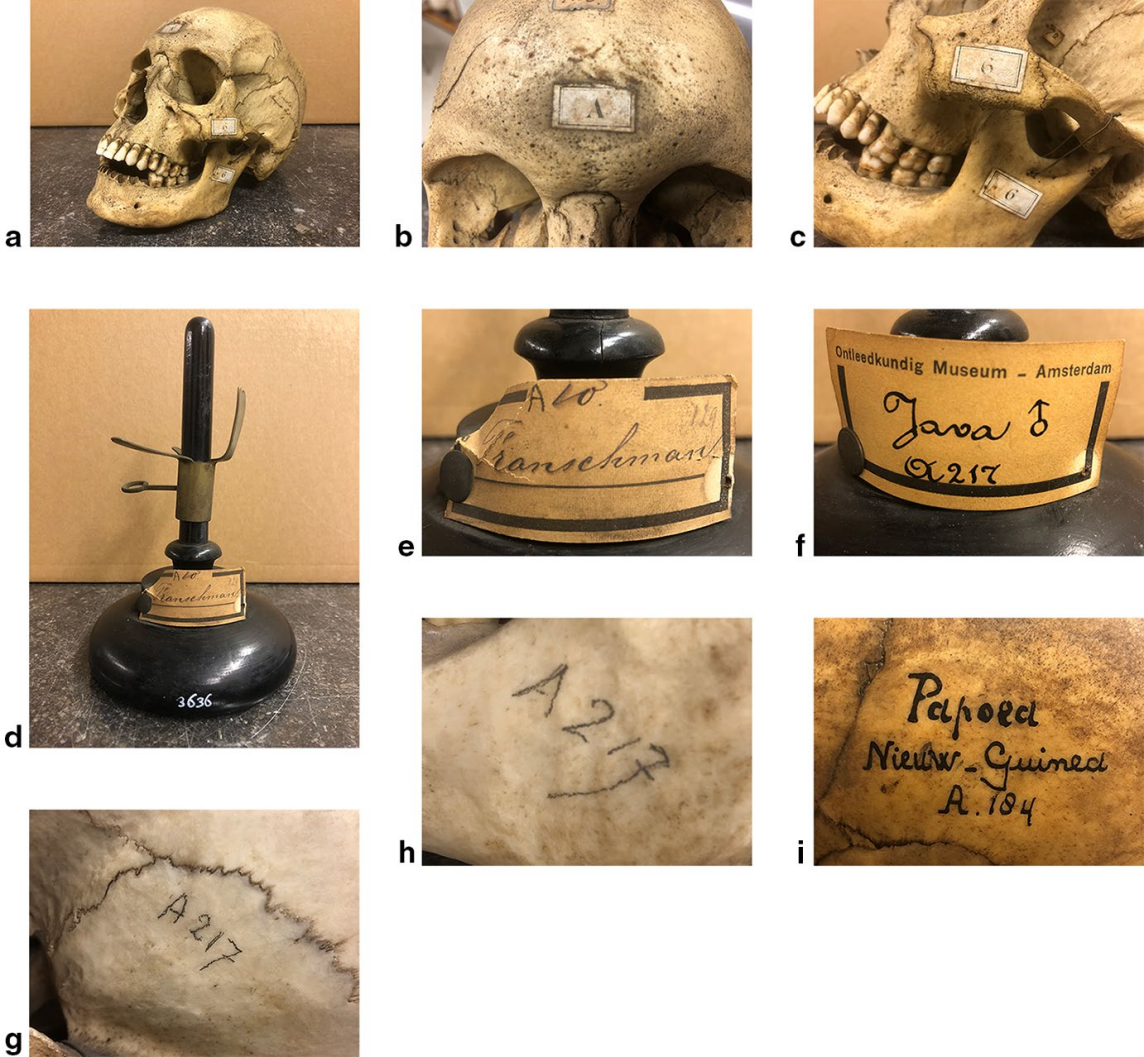
Four different styles of the Vrolik ‘A-number’. **a–c** skull of white U.S. American, Vrolik nr. A6, original labels by Willem Vrolik; **b** ‘A’ on frontal; **c** ‘6’ on zygomatic and mandible. **d–e** oldest label style on stand (of skull of Frenchman, Vrolik nr. A10); handwriting probably by anatomists Willem Berlin (1865–1879) or Lodewijk Bolk (between 1898 and 1909), (see paragraph 5.1). **f–h** label on stand and handwriting on skull (**g**: temporal; **h**: mandible) of Javanese man, A 217, dating 1909–1930. Handwriting probably by Evert Scheyde, Louis Bolk’s technician; **f**: Calligraphy on label; **g**: ‘normal’ pencil inscription on temporal; **h**: idem on mandible. **i** inscription in ink on parietal of a Papuan, A184. Probably by Mr. Boon, A. de Froe’s technician, dating 1943–1958

**Fig. 2:**
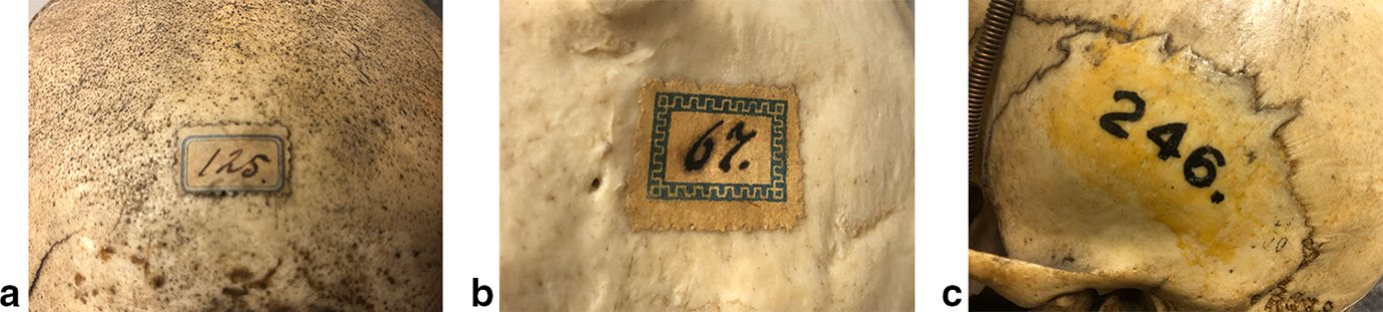
Two categories of more recent numbers in Vrolik’s ethnographic craniology collection. **a** and **b**: labels with numbers attached to frontal (left) or occipital (right) of all ‘racial skulls,’ written by or supervised by Lodewijk Bolk, ca. 1909 (see “The Normalization of Skulls of White People: Lodewijk Bolk” section); **c** stamped number by Arie de Froe 1938, on skull of a French person (Vrolik nr. A9), corresponding to a card index box containing measurements of 345 skulls, mainly from Amsterdam (see “No End to Normalization and Racialization: 1930–1960” below).

The A-number is the primary number identifying the skulls. Over time, these numbers have been (re)written on the skulls and/or on labels on wooden bases in at least six different styles of handwriting and labels (Fig. 1). Three other number categories can be found on the skulls. Two of these date from the beginning of the twentieth century, well after the 1865 catalogue, and will be discussed later in this paper (Fig. 2).

**Fig. 3:**
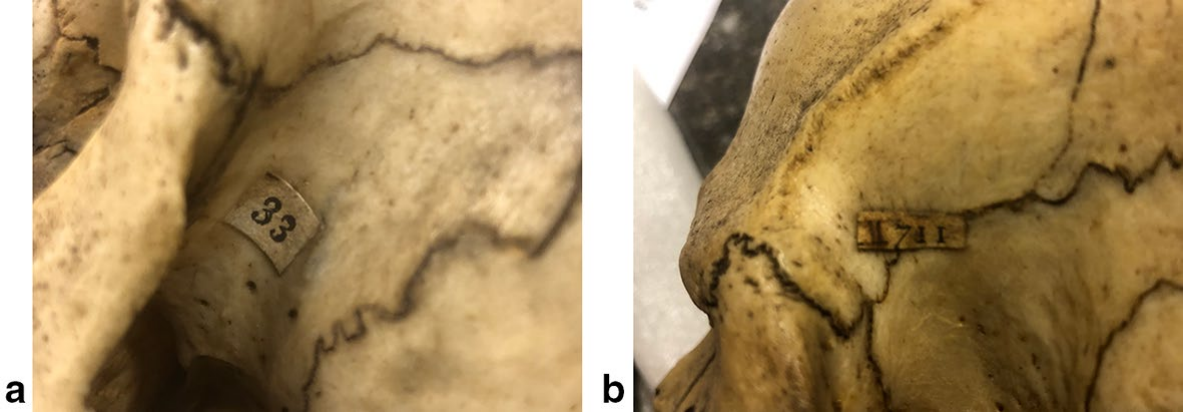
Numbers of Vrolik’s first and second (‘II’) collection. **a** and **b**: examples of labels of Vrolik’s original numbering system – ‘first’ and ‘second’ (‘II’) collection. **a** nr. 33, example from the ‘first’ collection on the skull of an English woman (later relabeled as Vrolik A4); **b** nr. II 711, example from the ‘second’ collection on the skull of a Russian man (later relabeled as Vrolik A59)

The final category of skull numbers (Fig. 3) occurs on all anatomical preparations of the Vrolik collection, not just ethnographical skulls. These labels are relatively small. On the skulls, they are usually glued to the left temple. These numbers come in two types: with or without a handwritten ‘II’. They are the original numbers of the Vrolik collection, before Dusseau’s catalogue, given to the collection during the lifetime of Gerard and Willem Vrolik.^8^ A catalogue of these numbers is missing.^9^

Close study of these numbers demonstrates that the collections with or without ‘II’ differ: all specimens without ‘II’ have a systematic arrangement, whereas those with ‘II’ lack such an arrangement. Numbers without ‘II’ reflect a systematic catalogue which no longer exists. The numbers with ‘II’ are additions to that catalogue, numbered chronologically. As such there exists a first and second Vrolik collection, the latter consisting of the additions to the first.

Based on the combination of known intervals for acquisition of specific specimens (exact dates are rarely provided) from literature or the Dusseau catalogue, as well as numbers from the second (‘II’) collection, it is possible to reconstruct that collection’s development through time. It is also possible to estimate the interval of conception of the first collection’s catalogue: ca. 1819–1826. That latter year is relevant because it is also the year in which the first written reference to the racial collection of the Museum Vrolikianum was published by Gerard Vrolik.

## Skulls of National Types: Gerard Vrolik

In 1826, Gerard Vrolik published his *Beschouwing van het verschil der Bekkens in onderscheidene Volkstammen* [*Consideration of the Difference of Pelves in Different human races*]. This publication has received interest from historians of race, as Gerard was the first to conduct a comparative study of racialized pelvises (Schiebinger 1993, p. 158; Hondius 2014, pp. 177–181).^10^ Gerard compared the pelvises of people of European, African and Javanese descent. His main references were Camper (1791), Sömmerring (1784), and Geoffroy-St. Hilaire and Cuvier (1819). Gerard largely followed Sömmerring and Cuvier in their conviction of racial hierarchy, especially their articulation of white, European superiority and black, African inferiority, making Gerard Vrolik among direct contributors to scientific racism.^11^

Gerard wrote that although he was convinced that African people in general stood on a “very low level of humanity (..) the Negro may count himself much higher above the ‘Boschjesman’ than the latter above the irrational beasts” (Vrolik 1826, p. 15). Nevertheless, despite this assumed hierarchy among the races, Gerard wrote that “even” African races were “not immune to civilization, ... if brought under favourable conditions” (Vrolik 1826, p. 16). But he doubted that they would reach the attainments of European civilization (Vrolik 1826, p. 6). This also corresponds with the fact that Gerard nowhere condemns slavery, whereas e.g. his contemporary anatomist, Friedrich Tiedemann proposed abolition (Tiedemann 1836 and 1837; Mitchell 2018). Gerard Vrolik’s stance on slavery aligns with his social position: he was part of the early-mid nineteenth century Dutch medical and scientific elite, confessionally Lutheran and politically conservative.^12^ In this sense Gerard’s position aligns with what Dienke Hondius describes as the dominant enlightenment form of “white supremacy in the Protestant Atlantic world,” interpreting races as human varieties in a broad monogenist “family of man” perspective but including a “strong self-evident notion of black inferiority and white superiority” (Hondius 2014, pp. 127–128). Likely, Gerard did not differ from most other racial scientists of the time in the conviction of monogenism. Although he is not explicit about the matter, indication of his monogenism comes from the arrangement of the ‘first’ collection numbers.

The first collection consists of about 50 racial skulls (see Table 1). The first of these are Dutch skulls, followed by skulls from neighboring countries. After skulls from other European nations, skulls from other continents were listed, first from Russia to Asia, and then from Africa (from south to north) to the Americas. The arrangement was geographic rather than racial, which is especially clear from the American skulls: the skulls of an African American, white American and Native American followed each other. Another clue is the position of a skull labeled “Hindu,” which follows skulls from China and precedes those of Javanese origin. If Gerard had followed the by then popular five-part racial classification of Blumenbach, based on craniological typology, this would have superseded geography: the skull from a “Hindu,” as well as that of the white American, would rank among Caucasian skulls.

**Table 1:**
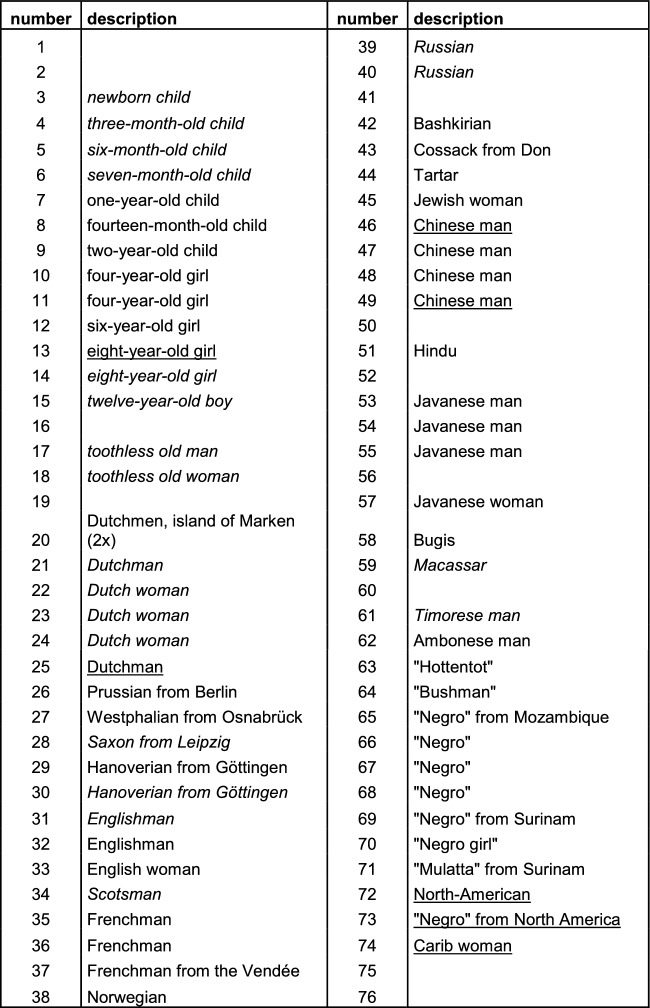
Gerard Vrolik’s ‘first’ collection

Names in italics: based on Tiedemann (Tiedemann ) only—skull no longer present in collection; names underlined: based only on skulls present in collection (skulls not mentioned by Tiedemann); other: skulls still present in collection and mentioned by Tiedemann

Gerard Vrolik is not unique in non-racial ordering in post-Blumenbachian racial anthropology; his teacher, Leiden anatomist S.J Brugmans, used a comparable order. Brugmans’s classification followed lines of latitude, emphasizing the view that these were formative of physical characteristics, including skull shape.^13^ Gerard apparently opted for a division following cardinal points (North–East–South–West). Had he been convinced by polygenism, this may have been apparent in his cranial classification, emphasizing race over geography, but this is not the case.

*Consideration of the Difference of Pelvises in Different human races* is Gerard’s first and only publication in racial anthropology. Likely, his interest in pelvises and race derived from his obstetric work, which is suggested by the systematic classification of the first collection with a hierarchical conception of anatomy. In this hierarchy, racial pelvises and the skulls are embedded within a broad collection of human and animal anatomy. Skulls, associated with brains and intelligence, took a primary position in this hierarchical arrangement. The pelvis, associated with organs of generation, was more basal. In this list, pelvises were separated from skulls by some 1000 entries, and racial pelvises were grouped together with series of children's and abnormal pelvises. The racialized pelvises are classified here obstetrically first, racially second, as variations of supposed normal pelvic anatomy.

Similarly, the racialized skull series starting with Dutch crania was preceded by a developmental series of (Dutch) children’s skulls (see Table 1). Here, too, the classification is rooted with reference to supposed normal anatomy. However, Gerard did not thereby necessarily imply that the Dutch were the most superior. Rather, other nationalities were embedded in an anatomical collection predominantly of Dutch origin, following as variations of an anatomical norm.

What placement of both racial crania and pelvises in the first collection demonstrates is that around 1826, anthropology (or ethnography) was not yet a separate category in Gerard Vrolik’s collection. This integration suggests that anthropology as a separate discipline had not yet been established and that its racial framework was still integrated into broader anatomical collections, typical of the eighteenth century and early nineteenth century.^14^

With skulls from Suriname, South Africa and Indonesia, most non-European skulls reflect the (expanding) colonial exploits of the Kingdom of the Netherlands in the first quarter of the nineteenth century, and Gerard’s social position within this colonial network. Notably, some donors were central governmental, legal and military figures of the Dutch colonial apparatus.^15^ The high percentage of skulls from Europe [27 out of 56] reflects the relative accessibility of European skulls; perhaps reflecting military conflicts in the Northern and Southern Netherlands in that period.^16^ The high percentage of skulls of different European nationalities also indicates a connection between typical national (and regional) characters and identities as these were constructed and refined through war, liberation and intermarriage of royal families in the first quarter of the nineteenth century. These characters were then linked to specific or typical cranial forms, as was assumed in studies of physiognomy and phrenology [cranioscopy]. This is also demonstrated by Gerard’s library, which counted about sixty titles on the topic *histoire naturelle de l’homme*, with keywords including: *des races humaines; cranioscopie; physiognomie* (Muller 1860, pp. 36–38).

Such specificity is less apparent in the non-European skulls. To Gerard, the specific nationality of the European skulls equaled the broader racial identity of the other skulls (Javanese, Chinese, “Negro” etc.). The “Negro” skulls are the most generally indicated, as their specific identity only indicates their blackness, but not geography (unlike, e.g. Javanese and Chinese). Moreover, excepting Dutch skulls, skulls classified as “Negro” form the largest group of skulls with the same identity in the first collection (viz. 7).

## Early Physical Anthropology: Willem Vrolik

After his 1826 publication on pelvises, Gerard Vrolik never again wrote about anthropology. Nevertheless, the *Museum Vrolikianum*’s anthropology collection expanded: the number of racial skulls in 1863 was 220—quadrupling the first collection’s size.^17^ Publications do not show that Gerard’s son Willem Vrolik was especially interested in anthropology. Willem Vrolik published only nine pages of research on the topic (Vrolik and Van der Hoeven 1859; Vrolik 1862). Almost exclusively, his research concerned teratology and comparative anatomy (e.g. Vrolik 1834, 1841, 1844–1848, 1852).

However, attention to racial skull acquisition from the second collection numbers (see Table 2) shows a different picture: both absolutely and relatively, the collection of racial skulls grew significantly in the last 17 years of Willem Vrolik's life, both regarding donated and purchased skulls. This growing racial-anthropological focus is further indicated by the contents of Willem Vrolik's library. The catalogue of his library contained 214 titles on anthropology, ethnography and colonial travelogue (Muller 1865). Of these, some 110 titles were about anthropology and ethnology proper, of which 71% were publications from after 1850, the majority after 1855.^18^

**Table 2:** Acquisition of Vrolik collection based on second collection numbers

	Interval	Total collection	Racial skulls^a^	Donated	Purchased^b^
col I (?1800-?1826)	?27 yrs	2100	56 (3%)		
col. II (< 1847)	?20 yrs	2000	52 (3%)	47 (90%)	5 (10%)
coll. II (1847–1863)	17 yrs	1000	136 (14%)	96 (70%)	40 (30%)
Total number		5100	270 (5%)		

^a^Racial skulls: this includes the crania, plaster casts of crania and plaster busts/face masks that are part of the craniological sub collection of the ethnographical collection. In total 270 entries; it excludes the 47 racial skeletons, pelvises and other parts. Of the total 270 entries, 26 could not be traced to any of the three intervals

^b^Purchased: this includes purchases from dealers, but also at auctions. It also includes 10 copies of plaster busts from the Musée d’Histoire Naturelle that Willem Vrolik received in exchange

The acquisition of the skulls, related books and other anthropological items such as plaster busts and casts demonstrate Willem Vrolik’s interest in this field starting in the 1850s. In this light, his choice to start compiling his catalogue by describing his anthropological collection is no coincidence. Willem had gained international fame in comparative anatomy and teratology because of a publication record from the early 1830s, but is anthropological research was cut short by his death in 1863.

Willem’s position as rising anthropologist is further indicated by his invitation to the “first German craniometric agreement,” a 1861 meeting organized by Moritz Wagner and Karl Ernst von Baer in Göttingen (Hoßfeld 2016, p. 96; Von Baer and Wagner 1861). Moreover, in 1859, Willem arranged the transfer of skulls from Indonesia to the *Museé d'Histoire Naturelle* in Paris. The skulls came from Willem Vrolik’s main supplier in Java, Cornelis Swaving.^19^ In exchange for the 35 skulls that Willem provided to Paris, he received 10 copies of racial busts from the *Musée*’s extensive collection of plaster casts.^20^ This exchange consolidated his position as an internationally recognized anatomist-anthropologist.

### Catalogue Partie Ethnographie 1860–1863

Willem Vrolik’s *Partie Ethnographie* is an expression of both the so-called old and new anthropology. Willem bridged Gerard’s typological collection of national skulls (and other such collections of single skulls from around 1800) and the craniometric averages of the later 19th and early twentieth century.

Arguably, the ‘old’ is represented by perceived completeness: the collection contained representatives of all racial categories from across the world. As such, the *Partie Ethnographie* is an encyclopedic global racial overview centered around racialized skulls and plaster casts.

Simultaneously, the *Partie Ethnographie* is an articulation of the so called new anthropology given Willem’s grouping of all race-related anatomical specimens together in one (sub)collection: not only skulls, but also plaster busts, casts, skeletons and pelvises. The pelvises were thus removed from their prior obstetric context to fit within a racial framework. The racial skulls were separated from the broader context of cranial development and variation to become the most prominent part of the *Partie Ethnographie*. Willem situated ‘race’ as an independent, primary organizing principle.

However, within the ethnographic sub-collection, Willem retained comparative anatomical hierarchy. He began with descriptions of all racial skulls, followed by pelvises and then skeletons. As in Gerard’s list, skulls came first. Following the theories of Blumenbach and Buffon, they were claimed to show “essential differences” of races related to “character, the kind of life, the conditions in which men live” (Dusseau 1865, p. 3).

That Willem began with skulls was an indication of craniometry’s prominence for mid-late nineteenth century physical anthropology. Relatedly, Willem recorded a large number–22– of different measurements for each skull. A further clue to the racial-anthropological primacy of the skull over other phenotypic features (eg. hair or skin colour) is visible among the plaster busts in the *Partie Ethnographie*. These busts were, rather unusually, not painted. This possibly indicated to Willem that their relevance was primarily in morphological racial features, rather than colour.

Willem Vrolik's racial classification drew from Blumenbach’s classification into five main types. In four of these types, Vrolik followed Blumenbach’s nomenclature: Caucasian, Mongolian, Malayan and American. The Aethiopian type he referred to as “Negro type” (Dusseau 1865, p. 54), possible explanations for which are discussed below. Willem’s overall order is: Caucasian—Mongolian—American—“Negro”—Malayan. It is unclear why he chose this specific arrangement, which does not match Blumenbach’s.^21^ Each type is taxonomically subdivided into family, tribe, nationality, region or people. Most of these categories and subcategories include a general description—in most cases well annotated—containing historical, zoological, linguistic and (stereotypical and prejudiced) characterological descriptions of each group and its environment.

Although the overall order of the catalogue arguably indicates otherwise, Willem occasionally hints at a racially biased hierarchy positioning the white people highest and the dark-skinned people lowest.

First, this hierarchy is taxonomically visible: racial and national cranial subdivisions are differentially articulated (Table 3). Most differentiated are subdivisions of Malayan and Caucasian skulls; this was likely a result of both Willem’s access, given his placement in the Netherlands, with colonial ties to the Dutch East Indies, and the increasing specialization and taxonomic granularity of developing racial anthropology. Comparison between the first and second collection’s additions shows that the number of European skulls had not increased markedly, while those from the Dutch East Indies had.^22^ This includes skulls classified as Malay, Indonesian Chinese, and Papuan. Like the skulls from Gerard’s first collection, most of these Indonesian additions reflect the state of Dutch colonialism at that time, including skulls from the Sulu islands, Borneo and from among the earliest expeditions of Dutch naturalists to New Guinea. Another difference between the first and second collection regards a shift from governmental, legal and military suppliers to medical and scientific sources. Fifty of the additions were from medical doctors in Indonesia, most notably Swaving. This reflects the professionalization of physical anthropology and the importance of “trustworthy” (ie. medically, scientifically trained) donors (Sysling 2015b, pp. 198–199), rather than merely influential ones. It also reflects Willem’s possible scientific specialization in the anthropology of Indonesia and Papua, evidenced in his paper on Alfurians and Papuans (Vrolik 1862) published shortly before his death in 1863.

**Table 3:**
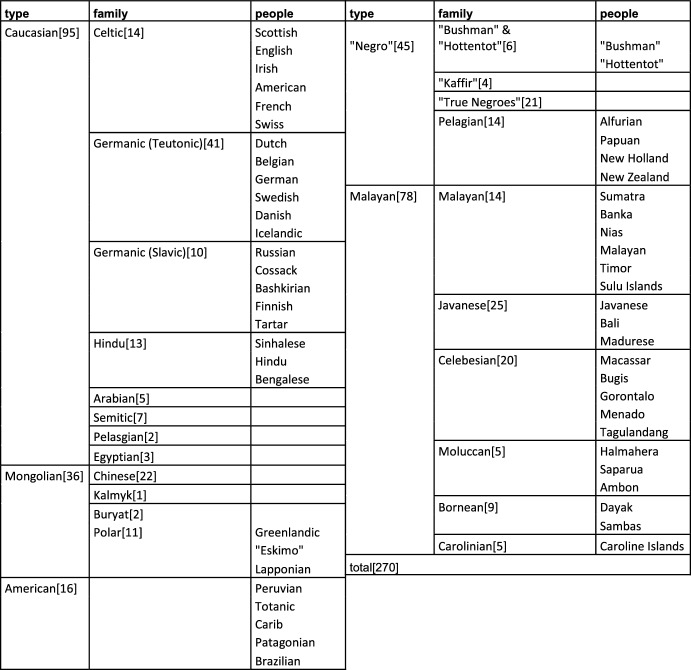
Taxonomy of Willem Vrolik’s *Partie Etnographie* (W.Vrolik/Dusseau 1865)

The types called “Mongolian,” “American,” and “Negro” are more crudely differentiated into sub-categories, with a smaller total number of skulls in these categories. Excepting skulls of Chinese people from the Dutch East Indies, Willem purchased almost all skulls from the Mongolian and American types. The Mongolian type skulls are still subdivided by family, but seldom further differentiated. The American type skulls are not even classified into families. The skulls of the Negro type have been differentiated into some detail, but only for inhabitants of South Africa and of New Guinea and surrounding islands, a reflection of Dutch colonial projects. Simultaneously, Willem’s inclusion of Papuan skulls—categorized as “Pelagian [oceanic] family (Dusseau 1865, p. 65)”—within the “Negro type” may also be one of the reasons for not using Blumenbach’s term Aethiopian for this grouping. That only some skulls representing this race originated in Aethiopia (a Greek, classical label for Africa) weakened the geographical designation in favor of a racial term fixed to the body: “Negro.”^23^

Although the highly differentiated taxonomy of skulls from the Dutch East Indies were apparently a focus of Willem’s interest, contrasting expressions of racialization may be strongest between the very generalized “family” of “True Negroes" (Dusseau 1865, p. 59) and those of the different European “families” of the Caucasian type, especially in relation to the characterological qualities attributed to these groups.

All people between Sahara and Kalahari and their descendants in the global African diaspora are classed here as one so-called family: that of the “True Negroes.” No other people group living in such a vast area were as taxonomically undifferentiated as this “family.” As already described in the discussion of Gerard’s first collection, “Negro” was the only purely racial categorization within the list, having no national, geographical or linguistic reference. Willem’s classification racializes even further: the term appears as well as a race (“Negro type” instead of Aethiopian type), so-called family (“True Negroes”), and specific determination (“skull of a Negro..” etc.). Willem’s singular use of the qualifier “true,” replicates the dehumanizing language of botanical and zoological taxonomy, and is applied to no other racialized group.^24^

In contrast, skulls of European “families” were more elaborately categorized, reflecting Gerard’s detailed list of skulls of national variations. (see Table 1). Like Gerard’s list, Willem’s catalogue started with a white European norm. However, there are notable differences. Willem’s racial categorization is based on linguistic and (classical) historical knowledge. He first describes all Indo-European “families” from west to east. In this arrangement, he did not start with his father’s supposed anatomical norm, i.e. Dutch skulls; the Celtic skulls came first. And Celtic meant both nations speaking a Celtic language and those known to have done so in the past (Britain, France, Switzerland), including skulls from white North Americans. Thus, white Americans changed position, from the back of Gerard’s geographic arrangement and to the front in Willem’s racial one.^25^

Contrast between generalized “Negro” and highly differentiated European nations extends beyond taxonomy and into characterological descriptions. Among European skulls, national characters connected to national skulls are the most extensively articulated and generally positive. Black people on the other hand, are described in sweeping and mostly negative terms. Although Willem writes that, “Negroes have intellectual powers greater than those generally ascribed to them” (Dusseau 1865, p. 59), he adds that:

there is no doubt that their sensuality is strongly expressed, that their physical powers are enormous and that they resist physical pain and morbid causes much better than the Europeans. But in all this, we must not forget that the Negroes in a state of slavery have been treated so badly by the Europeans that their morals and character have been perverted. (..) It remains to be seen what the impact of emancipation will be on their morale and their minds. Time will tell (Dusseau 1865, pp. 59–60).

Willem seemed pessimistic about the abolition of slavery in Atlantic Dutch colonies, which occurred on July 1, 1863, months before his death. He agreed that enslaved people were treated badly, but he emphasized the effects of this abuse, namely supposed moral degeneration and perversion of character, rather than argue for abolition. These views on slavery aligned with those of the conservative, Protestant (Lutheran) and elitist class to which Willem, like his father, belonged.^26^

Like Gerard, Willem expressed racist views regarding Black people, couched in the language of anatomy. He lists physical characteristics and repeats his father’s and Sömmerring's claims that these morphological details of Black people express a “tendency towards the shape and structure of beasts [‘brutes’]” (Dusseau 1865, p. 59). He added measurements from zoologist Jan van der Hoeven to argue that “the skull of the Negro, as the vessel of the brain, is smaller than that of the European” (Dusseau 1865, p. 59; Van der Hoeven 1842).

Further specification of Willem’s construction of bodily and mental hierarchy between black and white is found in description of skull no. A 169, the skull of a white girl from Amsterdam, 19 years old. Following craniological form, the skull is classed with the skulls of Black people. Willem wrote: “[T]here is no Negro blood in the family, and the young girl was no idiot. Her skin was very white” (Dusseau 1865, p. 64). With allusion to “idiocy” (a then-common medical term), Willem articulated a racist assumption of black intellectual inferiority. But, at the same time, he claimed that this skull demonstrated no such direct connection.^27^

The matter of the mind’s relation to brain and skull morphology may be the fundament of Willem’s anthropological interest, rooted in his comparative anatomical research. Such research already brought him close to debates about anatomical similarities and differences between humans and apes, and about the transformation or fixity of species. Willem was convinced of the fixity of species.^28^ In 1849, he published a study comparing the brains of chimpanzees, orangutans and humans, of which he included a newborn and “idiot,” arguing that ape brains were on a markedly lower level of development than those of humans (Vrolik and Schroeder van der Kolk 1849).^29^ Although this study did not address race explicitly, racial science was clearly connected. Tiedemann’s *On the Brain of the Negro, Compared with that of the European and the Orang-Outang* from 1836 may have been an important inspiration.^30^ Tiedemann’s aim was to prove that the brains of “Negro” people were no more simian than those of white people. Willem Vrolik shared much of Tiedemann’s anatomical and embryological concepts.^31^ In 1853 Willem would conclude: “[T]here may be some similarity in general terms [‘ín algemeenen grondvorm’], but the (..) opinion of the origin of the human race from that of the Apes is nonsensical and unsustainable [‘even onzinnig als onhoudbaar’], and that not even a Negro and Hottentot can be considered as an intermediate (Vrolik 1853, p. 156).” Although his words evidence his hierarchical racial thinking, humans were one species in his classification. Willem ends this section with a quote from a letter from Queen Gallitzin of Münster to Sömmering: “By the way, let it be printed again in detail in all scholarly and other newspapers that Moors are not apes, but humans, and only those whites who do not treat them as brothers are apes” (Vrolik 1853, p. 156).^32^

Like Gerard, Willem did not explicitly articulate a conviction of monogeny, but the above suggests that he held this view. The skull of the 19 year-old white Amsterdam girl is another clue: listing this skull among the crania of Black people would be impossible for a polygenist.^33^

## The Normalization of Skulls of White People: Lodewijk Bolk

After the collection was donated to the Athenaeum Illustre in the mid-1860s (University of Amsterdam since 1877), it was occasionally supplemented with skulls. In 1909, the anatomist Lodewijk Bolk inventoried all racial skulls in this collection; of the roughly 300 racial skulls, two-thirds were still from the original Vrolik collection.

Bolk became interested in anthropology around 1900, mainly focusing on the anthropological composition of the Dutch population (Bolk 1904; De Rooy 2011, pp. 208–245). In Bolk’s inventory, labels were attached to the forehead of the skulls. The inventory is now missing, so only the labels offer insight into the classification of the skulls circa 1909. From the reconstruction of that list from the numbers on the labels, no elaborate racial classification is evident. At most, there is a division into six main groups: African (and African descent), Malay, Caucasian, Mongolian, Papuan (Melanesian) and American. Unlike his predecessors, Bolk recognized at least six main races. The dark-skinned people of south-east Asia and Melanesia had split from those of Africa and African descent.

Bolk was an evolutionary morphologist in the tradition of Ernst Haeckel and Carl Gegenbaur (De Rooy 2011, pp. 52–91). Haeckel produced a phylogenetic hierarchy of the human races, referring to them as (hypothetically) separate species, placing Caucasians at top and darker people (Papuans, Africans and Australians) at the bottom (Richards 2008, pp. 244–255).

Bolk was similarly convinced of human racial hierarchy in which “cultured peoples” [“cultuurvolkeren”] ranked higher than “natural” or “primitive peoples” [“natuurvolkeren”; “primitieve volkeren”], to which he ascribed “Negroes” and Papuans (Bolk 1905, p. 358; 1915a, pp. 390–391). Like Haeckel, Bolk suggests that these races may be distinct species: he refers to them as “human races or species that currently populate the earth,” of which “the white man is at the zenith of development, leaving the rest of the human species [‘menschensoorten’] behind” (Bolk 1912, pp. 188–189). Bolk’s evolutionism aligns more closely with polygenism than the nineteenth century pre-Darwinian racial conceptions of father and son Vrolik.

Bolks scientific racism only grew more extreme in the 1920s, likely in part due to the rise of socialism (and communism) in the late 1910s. Bolk, who viewed himself as a liberal (Gould 1981, p. 150), considered socialism a “chimeric and unnatural principle [‘hersenschimmig en onnatuurlijk beginsel’]” (Bolk 1918, p. 11). In 1926, he concluded that it “should make us lament that the social development (or degeneration?) of the last decades has facilitated a ‘pénétration pacifique’ of the Negro race in the centers of culture in Europe” (Bolk 1926, p. 2337).^34^

### White is Normal

Bolk’s hierarchical concepts of cultured and primitive led to the de-racialization or normalization of the Vrolik’s European skulls and the further exoticization or othering of skulls of other races. This process only emerges by attention to the skulls themselves, as it is not present in published literature. In the period between roughly 1909 to 1930—from the move to a new laboratory until Bolk’s death, a large part of the original Vrolik collection was renovated, including new jars, mounts, stands, and labels. The most recognizable trace of this renovation is handwriting on these new labels—likely from Evert Scheyde, the lab’s technician between 1904 and 1938 (see Fig. 1d–f).

When considering the renovation of Vrolik’s collection, focusing on which parts of that collection were renovated and which were not offers insight into what was relevant from the 19th-century collection to an early 20th-century anatomist. Many of Vrolik’s ethnographical skulls were renovated, apparently relevant to Bolk. Willem Vrolik’s labels were scratched from the forehead, cheekbone and jaw, original varnish was removed, and new labels with curly calligraphy were pinned to the stands. Of the ethnographic collection, only the European skulls were not renovated. The choice of whether to renovate based on the origin of the skulls shows their relative relevance for Bolk’s research or museum presentation.

The only skulls of European origin that were renovated in this period were Dutch skulls from the former Zuiderzee islands, suggesting that they had kept or gained anthropological value, likely concerning Bolk’s research into Dutch origins. Residents of these isolated islands were thought to represent the most original and supposedly racially pure Dutch form (De Rooy 2011, pp. 208–233).^35^ Bolk treated the remainder of Vrolik’s European skulls as anthropologically less relevant. Their provenance was not detailed enough to render them proper samples for at the time modern physical anthropological study. They did not come from an isolated location or, when specific provenance was known, the sample consisted of too few skulls per location to correct for individual variance.

Apparent neglect of the European skulls from the Vrolik collection may indicate part of a process in which these skulls changed their meaning within a collection, having transformed from skulls of national types (Gerard), to comparative racial skulls (Willem), to supposed normal skulls (Bolk).

### Foramen Magnum and Fetalization

Between 1909 and 1925, Bolk developed his fetalization theory of human evolution. This theory was largely a response to Ernst Haeckel's recapitulation theory, and, like that theory, made a strong link between embryonic development and evolutionary descent. Bolk noted that in many characteristics, human bodies looked more similar to young rather than adult apes. Bolk concluded that during human evolution, fetal or juvenile traits were conserved into adulthood: “Man may be considered as a primate fetus that has become sexually mature” (Gould 1977, pp. 133–135 and 353–366; Gould 1981, pp. 148–150; De Rooy 2011, pp. 166–207).

Many characteristics that Bolk classed as fetal were craniological: the foramen magnum’s position on the occipital bone, the face’s position relative to that of the brain, the shape and position of the browridge, orbits and jaws, and the development of the teeth. In his study of the foramen magnum, Bolk included racial skulls, including those of Vrolik (Bolk 1915b).

Morphologists before him, like T.H. Huxley, had noticed that the foramen magnum’s position and angle differed among humans, monkeys, apes and other mammals. In humans, the position was more central in the cranial base than in other species, where it was located more posteriorly. Researchers ascribed this difference to human upright stature. Huxley also related the foramen magnum’s angle to prognathism: the more prognathous, the more oblique the position of the foramen magnum; not only among monkeys and apes, but also among human races. According to Huxley, examples of prognathous human races, eg. an Australian and a “Negro” skull, were demonstrative (Bolk 1915b, p. 668). The implicit conclusion was scientifically racist: another supposed proof of the inferiority of the Black people, indicating an evolutionary position closer to apes compared to orthognathous white people.

**Fig. 4:**
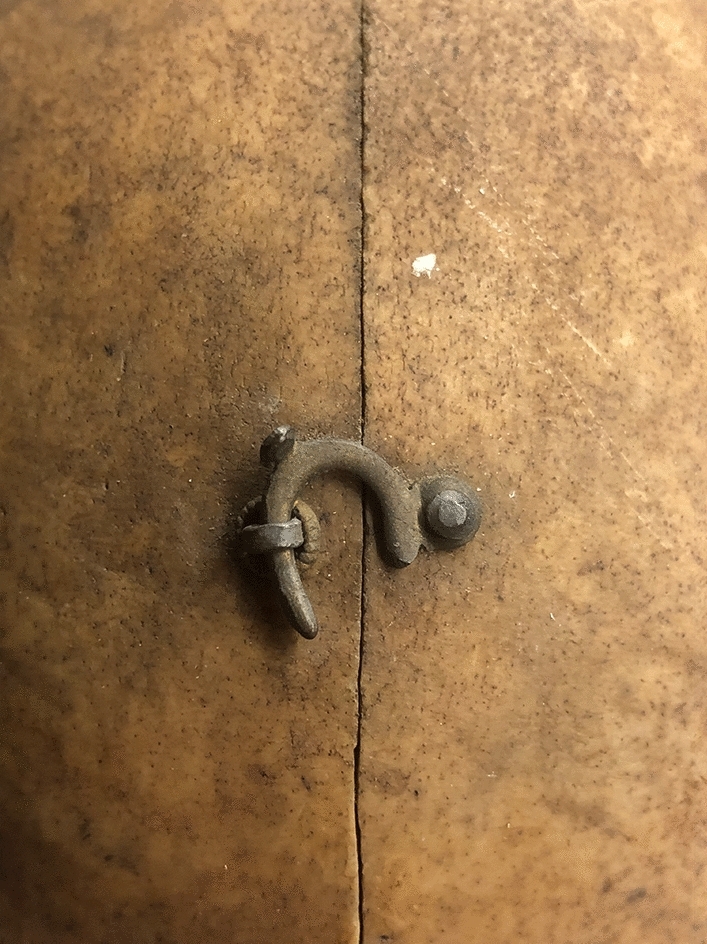
Racial skull, bisected mid-sagitally by Bolk, ca. 1915. Frontal bone of the skull of a Papuan man (Vrolik A184) demonstrating the midsagittal section with the two halves of the skull kept together by brass hook and eye. A second hook and eye was attached to the occipital bone of the skull

Bolk investigated the matter further. He sawed 133 skulls of monkeys and apes mid-sagittally, as well as 54 skulls of children and 50 human racial skulls (see Fig. 4). The latter comprised five groups of ten skulls each of Javanese, Zeeland, Papua, “Negro,” and Frisian.^36^ Of the racial skulls Bolk prepared, at least 22 were from the Vrolik collection.

Bolk’s selection of racial skulls demonstrates his scientific methodology and concepts about race around 1915. First, Bolk selected only non-European Vrolik skulls. The European skulls he used were Frisian and Zeeland skulls from his own collection.^37^ Second, of these non-European skulls, he used only those consisting of more than just one or two within each grouping. In this sense, Bolk’s methodology was fully aligned with the anthropological methodology of his time, requiring a statistical average sample of a population rather than a few exemplars.

The skulls were cranial-racial representatives of types which Bolk did not mention but can be deduced: Javanese skulls represented the brachycephalic (shortheaded) and prognathous Malayan race; skulls from Zeeland signified the brachycephalic and orthognathous Alpine (Celtic) group of the “white race,” and Frisian skulls the dolichocephalic (longheaded) and orthognathous Germanic group of that same race. Finally, Papuan and “Negro” skulls, both prognathous and dolichocephalic, representing the “black races” of Southeast Asia and Oceania and of African descent.

In most of these cases, Bolk’s selection comprised a delimited and specific provenance: an island (Java, New Guinea) or an area (Friesland, Zeeland). This fit within a paradigm in which individual skulls from approximately the same place were necessary to create valid measurements of averages, even though these averaged selections would represent their entire respective major race.

As mentioned previously, within Vrolik's collection of Caucasian skulls there were not enough skulls of each nation or region to create reliable averages. This stressed the irrelevance of the Vrolik collection’s national Caucasian skulls for supposed modern anthropology.

If uncertain provenance was reason for exclusion of Vrolik’s skulls of white people, this was less crucial in selection of skulls of Black people. As Gerard and Willem Vrolik both had assumed before him, the generic designation “Negro” was sufficiently specific, and no further indication of provenance was required for study of these skulls. Bolk used nine skulls of this grouping from the original Vrolik collection, and one added more recently, but without any apparent concern that these came from Suriname, Guyana, the coast of Guinea, Mozambique, Amsterdam, or the Dutch East Indies. Had Bolk used the same criteria for his two categories of skulls of white people, he would have assembled a pan transatlantic mix of white English, American, French and Swiss skulls for the Celtic race, and German, Dutch, and Scandinavian skulls for the Germanic race.

Despite Bolk’s conviction of racial hierarchy and inferiority of “natural peoples” such as Black people of African descent and Papuans, his research on the foramen magnum did not demonstrate a difference in the position and angle of this hole in these or any other races. However, this conclusion changed nothing regarding the hierarchy of the races in Bolk’s fetalization theory (Bolk 1923). Bolk’s premise was that the more fetal features were preserved, the more supposedly human and superior a race was. The foramen magnum was perhaps conveniently excluded as an argument in this fetalized racial hierarchy, which instead focused on features like skin colour.

## No End to Normalization and Racialization: 1930–1960

Vrolik's collection of racial skulls, with Bolk’s additions, retained its relevance after the Second World War. In Dutch anatomical institutes, explicit racial studies within physical anthropology ebbed gradually in the 1950s and 1960s. Anthropological skulls were still collected at, for example, the (former) Colonial Institute (Van Duuren et al. 2007).

Yet another transformation of Vrolik's racial skull collection occurred between 1938 and 1958, by or under supervision of anthropologist Arie de Froe. In 1938, de Froe started his career as an anthropologist with a craniological dissertation on skulls from Amsterdam entitled: *Measurable Variables of the Human Skull and their Mutual Correlations Related to Age and Gender*.^38^ After WWII, he worked as lecturer in osteology and anthropology, and from 1953 to 1976 he was professor of anthropobiology and human genetics.^39^

In his dissertation, De Froe studied 345 skulls from the anatomical laboratory of Amsterdam which he marked with black stamped numbers (see Fig. 2c), corresponding to a card index box containing measurements and details for each skull. Known sex and age at death were necessary criteria for inclusion in the study. As De Froe wrote, most of the skulls came from Amsterdam inhabitants, collected between 1883 and 1909, mostly amassed by Bolk (De Froe 1938, pp. 6 and 9). But inclusion of nine skulls of Austrians from 1887 and five skulls from the original Vrolik collection shows that specific national or local origin was not essential: any skulls of white people of known age and sex were included. Skulls from the Vrolik collection in this series included a Swedish sailor (Vrolik, nr. A 40) and a French murderer (nr. A 9). The skull of the 19-year-old Amsterdam girl, grouped by Willem Vrolik among the so-called family of “True Negroes,” found a place in this exclusively white series. Stripped of their labels, these skulls became part of a kind of normal series, a reference collection of development and individual anatomical variation—for white people.

In 1958, an inventory was made of the entire collection of the Amsterdam Anatomical Institute. The reason for this inventory is unclear. Nevertheless, as Vrolik collection specimens were given a location number in this inventory, it offers perspective on the location of Vrolik's racial skulls in the Institute. In this inventory, most location numbers of the preparations and specimens were given the prefix “Mu,” for Museum (museum hall of anatomical laboratory). Some were given the prefix “AN,” a presumed abbreviation for Laboratory of Anthropobiology. All of these “AN” skulls have in common that their original (Vrolik) number and their race or origin are written in black ink on the parietal bone (see Fig. 1i) and their labelled stands were removed. By writing information on the skull itself, the stand was no longer necessary for identification; without stands, the skulls were more mobile and more easily useable for hands-on study.

Vrolik's racial skulls located in the department of Anthropobiology mainly consisted of skulls from present-day Indonesia: of the 34 skulls still present in the collection, 23 are Indonesian, including New Guinea. Including racial skulls from the Bolk collection and others, the selection consists of 109 skulls still present, of which 90 are from Indonesia, and of these 38 from Papua—one third of the total. This strongly indicates that the selection was to further research in physical anthropology of the former Dutch East Indies. It therefore seems unlikely that the selection was made by De Froe, who had no specific interest in that region. It is more likely that H.J.T Bijlmer had selected and inscribed the skulls. Bijlmer (1890–1959) was a private lecturer in physical anthropology at the same location between 1933 and 1956, largely overlapping with De Froe’s lectureship. Bijlmer had a strong interest in the anthropology of the Indonesian archipelago, especially Papuans (Sysling 2015a, pp. 133–155).

Regarding the selection: in most cases, more than two skulls of each category (race or area) were chosen, often even five or more. This number was apparently considered a sufficient sample size assuring supposedly objective determination of the craniometry of the specific race or type. This would explain why many other Vrolik collection racial skulls (including Indonesian skulls) were not included in the selection, as these skulls consisted of only one or two representatives of their category. They fit the museum rather than a racial study collection requiring large sample sizes. As such, they kept their typological status since they were collected for that purpose by Gerard or Willem Vrolik.

A small part of the anthropobiological selection (some 18 skulls still present) are not of the Malayan or Papuan race. They consist of two categories: skulls of Chinese and of Black people of African descent. Why are these skulls part of this study collection? As the Chinese skulls are all from Indonesia, their inclusion could have been relevant for comparison with skulls of ‘indigenous’ Indonesians. For the skulls of Black people of African descent, a similar purpose is more problematic. Perhaps they served as comparison to the skulls of Papuans, as both were considered “Negro?” Or did they serve to represent Belanda Hitam?^40^ Two of the skulls were Belanda Hitam, the others were from Suriname, Amsterdam, and the U.S. In any case, for this racial selection, it apparently did not matter that these skulls of Black people came from across the African diaspora. It seems that as with Bolk’s foramen magnum research and Willem Vrolik’s classification of the “family of True Negroes,” Black people of African descent were considered a uniform race. No European or otherwise Caucasian skulls were in this selection of racial skulls, a trace of the ongoing normalization and de-racialization of the European skulls—and people.^41^

In 1958, the registered number of European skulls of the original Vrolik collection was only 18. Many skulls were lost; classification as non-racial supposedly normal skulls in an anatomical collection meant that they had no additional value beyond a supposedly neutral anatomical range. Consequently, many of them were lost, possibly due to use for purposes in anatomical research and teaching: e.g. sawn in slices to demonstrate internal anatomy, or dropped by a careless student during a practical osteology class.

## Conclusion

As physical anthropology increasingly focused on anthropometry and craniometry, the collection of skulls from multiple individuals from a well-circumscribed area increased in importance. This new supposedly objective anthropology of averages replaced the old typological anthropology which demanded an overview of global human types, each represented by a few exemplars. This development is clear in the shelf life of the Vrolik collection of racial skulls from the 1820s to the 1950s. Absent in Gerard Vrolik’s national and typological skulls, the tendency towards quantification and objectivity becomes apparent in the 1850s in Willem Vrolik’s focus on craniological measurements and accelerated collecting. In this sense, Willem’s interest in racial skulls corresponds with the birth of supposedly modern^42^ physical anthropology. The growing relevance of skulls from Indonesia (and of larger numbers of skulls from medically trained donors) since the 1850s can certainly be seen as a further expression of the development of such a modern physical anthropology in Amsterdam entangled within Dutch colonial-imperial networks.

However, typological approaches persisted. With Willem Vrolik, this tendency emerges in the purchase of famous type exemplars, such as Botocudo and Māori, and the acquisition of plaster busts. In the period from around 1900, the value ascribed to most of these typological exemplars remained, as is seen by the fact that most were renovated from 1909 onward and were part of the museum exhibit in the 1950’s.

Two important further conclusions can be made regarding both typology and objectivity in the changing meaning of Vrolik’s collection of racial skulls. These concern the remaining relevance of the generalized racialization of the skulls of Black people and the disappearance of the racialization of the skulls of white people in anthropological study.

The Vrolik collection’s white European skulls faded as racialized specimens. Representing the physiognomy of European national characters, they were the most prominent part of Gerard Vrolik’s collection. Although Willem Vrolik hardly expanded this racial sub-collection, it remained an integral part of his encyclopedic collection of world races. After 1863, the skulls of white people in the collection became less relevant in the presentation of racial skulls. The only exception concerned Dutch archaeological skulls or skulls from isolated Dutch Zuiderzee islands. These were still considered racially relevant insofar as they were representatives of the supposed original Dutch.

For other European skulls, their de-racialization had profound consequences. As supposedly normal skulls, they were prime material for varied anatomical purposes. Some were included in developmental series, but the fate of many others is unknown. The Vrolik collection originally contained 57 European skulls. 40% of these (23 skulls) are now missing—more than any other group. As such, the changing meaning of the Vrolik’s collection of racial skulls shows that from the second half of the nineteenth century onwards, skulls of white people ever more equaled the neutral or the norm rather than a race at all. This gradual de-racialization of skulls of white people generally matches the unmarkedness of whiteness as part of a constructed white superiority mentioned in the introduction (Blakey 2021; Frankenberg 1994). However, the Dutch archeological skulls and skulls from isolated regions demonstrate a relevant exception. They were still considered from a clear racial perspective: one that concerned the supposedly original Dutch and as such representing the ancestry of this group of white people. This ancestral element demonstrates a relevant difference between the consideration of white people in a colonial or settler society like the US and that of various European nations.

For skulls of Black people in the Vrolik collection, the opposite of de-racialization occurred: from the early nineteenth century to the 1950s they retained racial significance based on the assumption of typological uniformity, although the skulls could not possibly be regarded as homogeneous regarding provenance. The concept of black uniformity starts with Gerard Vrolik with his use of the phenotypical non-identity of “Negro” as a category, as opposed to cultural–geographical identities based on nation, island or people in his geographic taxonomy. Similar assumptions structure Willem Vrolik’s taxonomy: although Willem’s arrangement of the five main races is non-hierarchical, four types follow Blumenbach’s geographical terminology. Black people are labelled with the non-identity of “Negro type.” And where the skulls of white people remain differentiated as separate peoples and nations, even among recent settler-colonists (white U.S. Americans), most skulls of Black people were lumped into the “family of True Negroes,” regardless of origins, whether Ghana, Mozambique, Suriname, or the United States. Furthermore, although both Vroliks seemed to be convinced of monogeny, both also ranked the Black people as inferior, primitive or anatomically simian.

In the following period, these skulls maintained racial-typological relevance even within supposedly modern physical anthropology. For Bolk’s studies on the *foramen magnum*, it did not matter that the skulls of Black people came from across the globe, if their provenance was known at all. The opposite was true for other groups: their well-determined provenance and specific, circumscribed range of origin was essential for scientific argument. Bolk could have decided to exclude skulls of Black people from study based on provenance standards he assumed for other samples—but he did not.

For Gerard Vrolik, Willem Vrolik and Bolk alike, the identity of a race or a people was based on biology (physical features) together with linguistic, geographical, cultural, and historical elements. Authenticity meant that homogeneity existed in biological, linguistic and cultural aspects of a people or race. The exceptions were the “Negroes.” Their identity was a non-identity based on biology only, centered around their blackness—but operationalized through skulls rather than skin color. Thus, the word “Negro” was at the same time a race, a people and a stereotype. For Black people, whether in diaspora or not, culture, language or history was irrelevant. This artifice of differential racialization made “Negro” the ultimate and enduring reference group for anthropology in the Amsterdam anatomical collection—the absolute and generalized other.

## Footnotes

1 See for biographical details about Gerard & Willem Vrolik & about Museum Vrolik: (Van der Hoeven 1859, 1863; Kruizinga 1963; Baljet 1983, 1984; De Rooy and Van den Bogaard 2009).

2 Catalogued by Willem Vrolik’s assistant Justus Lodewijk Dusseau. The collection was subdivided by Willem into five sub collections, each indicated by a letter (A, B, C, D and E); A for ethnography; B for osteology; C for splanchnology (organs),; D for pathological anatomy; E for teratology (congenital malformations). F is an appendix of animals in alcohol, mostly reptiles.

3 Unfortunately, hardly any correspondence to or from father and son Vrolik exists regarding the collection.

4 “Shelf life” or “collection life” is a term introduced by Fenneke Sysling to describe the object life of a specimen from the moment it enters a museum collection. See: Sysling (2015b).

5 The non-craniological part of the ethnographical collection of Vrolik originally consisted of only 47 entries (under 10% of the total). Except for pelvises, the non-craniological part is omitted from this paper.

6 See for the relevance of material culture for historical research and object biography: (Prown 1982; Appadurai 1986; Gosden and Marshall 1999). See for the object biography in relation to human remains in museum collections: (Alberti 2005; Roque 2010; Sysling 2015a; Huistra 2018; Hendriksen 2012; Claes and Deblon 2018).

7 I deliberately chose the word Science and not Pseudoscience, often used to describe racial anthropology (and fields like eugenics, phrenology, criminal anthropology, etc.). Although I fully agree that the studies of father and son Vrolik, Bolk and the like are not ‘scientific’ by present standards, they were treated as such in the past. This does not mean that I want to downplay the harmful consequences of this scientific racism in this way. Rather quite the opposite: scientific racism and racialism are unfortunately still part of the ‘toolbox’ of racists until today, and this article contributes to our knowledge about how firmly these stereotypical and hierarchical ideas are rooted in the science of the past.

8 Proof for this is found in publications by German comparative anatomist Friedrich Tiedemann (Tiedemann 1836, 1837). In demonstrating racial equality in brains and skulls, Tiedemann determined the volume of the cranial cavity of a large number of racial skulls from several European collections. But the number of skulls differ between the English version of 1836 and the German version of 1837. The German version counts more skulls and from more craniological collections. The skulls from Vrolik’s collection are only included in the German version, where he also included the numbers assigned to them, including the old Vrolik number.

9 Furthermore: due to the use, wear and tear and deliberate processing, renumbering and relabeling of the skulls and other collection, these numbers are only still present in approximately 33% of the Vrolik collection.

10 The Dutch word “volkstammen” could be translated as “tribes of people;” “human races” is based on Vroliks own words, for he simultaneously published this text in French, entitled: “Considérations sur la diversité des bassins de différentes races humaines.” Schiebinger (1993) cites the French version, but erroneously refers to the author as Willem Vrolik.

11 Vrolik, regarding the differences in anatomy between “Aethiopian and European” : “Sömmerring heeft er zeer velen opgeteld en met eene behoedzamen nauwkeurigheid beschreven... Ik mag echter bij de beschouwing van het bekken, niet onopgemerkt laten, dat ook daarin zonderlinge blijken voorkomen van het meer dierlijke bestaan des Negers” (Vrolik 1826, p. 6). Sömmerring drew the same conclusion: “[I]m allgemeinen, im Durchschnitt, [gränzen] die afrikanischen Mohren doch in etwas näher ans Affengeschlecht, als die Europäer...” (Sömmerring 1784, p. 32). See for discussions about the (un)intentional scientific racism of Petrus Camper & J.F. Blumenbach: (Meijer 2015, pp. 187–213; Schiebinger 1993, pp. 146–160; Hondius 2014, pp. 163–189; Junker 2019, pp. 96–112).

12 See for Gerard Vrolik's prominent social position in Dutch science and medicine: (Van der Hoeven 1859; Kruizinga 1963). As promiment Lutheran, Gerard was trustee of the Royal Dutch Lutheran Seminary (Amsterdamsche Studentenalmanak 1832, p. 36). For an insight in Gerard’s conservative perspective: Letter of Gerard Vrolik to Agnites Vrolik, March 24, 1848; Familiearchief Familie Vrolik; Nationaal Archief Den Haag, 2.21.172: 86. See also: Ray (2022, p. 127). Gerard Vroliks other son Agnites (1810–1894) was a conserative politician; see http://www.parlement.com/id/vg09lljk47yh/a_vrolik (accessed April 27, 2023).

13 See for Brugmans arrangement of skulls: (Sandifort 1827, pp. 35–40; De Jonge 2001, p. 24). On Blumenbach’s racial classification, see: Irvin Painter (2003, p. 16).

14 Gerard Vrolik was not unique in the embedding of racial skulls in broader anatomical collections: e.g. inventories of other contemporary Dutch anatomical collections such as Bosscha (Amsterdam), Sandifort (Leiden) and De Fremery (Utrecht) show such typical broad anatomical collections.

15 Skulls from Indonesia reflect the state of Dutch colonisation at the time, with especially Java and the Moluccas colonized and Sumatra and Sulawesi only to a very limited extent (e.g. Macassar and Bugis skulls from the southwest part of Sulawesi that was being colonized at the time). Examples of donors are: Governor-General of the Dutch Indies Godert Van der Capellen (1778–1848]), commander of the Royal Dutch Indies Army, leader of the Sulawesi expedition General Hubert de Stuers (1788–1861), and Attorney General of the Cape Colony Daniel Denyssen (1770/75–1855) (Dusseau 1865, pp. 55, 94, 105).

16 The French Revolutionary and Napoleonic wars (1792–1815) included some military campaigns into the northern and southern Netherlands, like the Anglo-Russian Invasion of Holland (1799) and the battle of Waterloo (1815), where many English, Russian, French, Prussian soldiers died. Gerard’s teacher Brugmans, who was Inspector General of the Military Medical Service collected many skulls and bones of soldiers who died in military field hospitals in several of such campaigns. See De Jonge (2001, pp. 24, 28–30, 38, 40). As the provenance of many of the European skulls of Vrolik's first collection is unknown, we cannot be certain that these battle grounds and hospitals were indeed also the sources for many of these skulls.

17 272 entries within the category crania in Dusseau’s catalogue; minus 19 plaster cast of crania (mostly from Anders Retzius) and 29 plaster busts/masks and two objects not related to craniology.

18 Following author’s review of the library catalogue (Muller 1865).

19 M.D. Cornelis Swaving (1814–1881) arrived in Batavia in 1842, rising to rank of first city physician in 1859. Besides collecting skulls for Willem Vrolik, he also had his own collection of more than 300 skulls ( Swaving 1861/1862; Blok and Molhuysen 1933).

20 “*J’ai reçu en effect (..), les quatre bustes parfaitement en ordre et je suis très content de la belle collection, que Mr. de Quatrefages m’a fuite à Paris” (Letter W. Vrolik to H. Jacquart, January 13, 1859); *I thank Veronique Laborde & Dr. Serge Bahuchet of Musee de l’Homme for providing the letter, originally found in the Archives of the Museum Nationale d’Histoire Naturelle. Willem received two busts of Africans, one from a Tahitian boy, three from Greenlandic Inuits and four from Icelanders—the last seven from Prince Napoleon’s polar expedition (Dusseau 1865, pp. 18, 45–46, 64, 120).

21 *Blumenbach considered the Caucasian type as the neutral, supposedly original form, all other forms were seen as derived from this. He did however consider an aesthetic hierarchy in which he deemed the Caucasian race to be the most beautiful (*Painter (2003, pp. 22-23, 31, 37; Hondius 2014, p. 169).

22 The first may be related to increase in skulls from the Indonesian archipelago since 1847. The ratio of Malayan skulls between coll. I, coll II (< 1847) and coll II (1847–1863) is 10:20:40; that of European skulls is: 27:10:6.

23 Willem Vrolik was not the only one to arrange skulls of Papuans, Australians and inhabitants of surrounding islands in the “Negro type.” See: e.g. (Von Baer 1859). Morton also ranked the Papua and Australians among the “dark skinned race.” In 1839 he called this race “Ethiopian” (Morton 1839, p. 7), but by 1849 he changed this to “Negro” (Morton 1849, p. 6). I thank Paul Wolff Mitchell and Lisette Jong for conversation regarding this point and Mitchell for information on Morton.

24 See Curran (2011, pp. 10–11), where this link with dehumanizing botanical and zoological language is also made.

25 Likewise, skulls from the Indian subcontinent were now also placed within the Indo-European group.

26 See: Van der Hoeven (1863) and note 12.

27 Watkins (2018 p. 36, note 7) describes similar cases from the craniological collection of T. Wingate Todd, from the notes of Todds student William Montague Cobb.

28 Like many other zoologists and anatomists during his life, e.g. Georges Cuvier, Richard Owen and his associates (e.g. Van der Hoeven 1861).

29 Willem Vrolik kept his anti-transformationist perspective after the publication of Darwin’s *Origin of Species* (e.g. Schroeder van der Kolk & Vrolik 1862, pp. 9–10). In 1860, the illustrated chimpanzee brain became an element in a debate about human evolution between evolutionist T.H. Huxley and anti-evolutionist R. Owen. When Willem was invited to Göttingen in 1861, he lectured about chimpanzee brains(Rupke 1995, pp. 274–279; Mehos 2006, pp. 62–63; Von Baer and Wagner 1861, p. 30; Hoßfeld 2016, p. 98).

30 As previously mentioned, Tiedemann contacted the *Museum Vrolikianum* for this study.

31 Willem Vrolik became acquainted with Tiedemann’s comparative anatomical work during his student-years in Utrecht, where the professor of Natural History, N.C. De Fremery, lectured with Tiedemann’s textbook (Van der Hoeven 1863, p. 5).

32 Letter from queen Gallitzin of Münster to Sömmerring (quoted from Wagner 1844, p. 78).

33 Also notable is the circle of renowned anthropologists that Willem referred to or was in contact with, most of which were outspoken monogenists: Blumenbach, von Baer, de Quatrefages and Tiedemann.

34 To reinforce this remark, and his conviction of the inequality of different races, Bolk quotes American eugenicist Madison Grant). Bolk's more radical racist perspective since the 1920s is further underlined by literature he added to the laboratory’s library. In 1918, this library did not contain A. de Gobineau’s *The Inequality of the Human Races* or Grant’s *The Passing of the Great Race*. Some ten years later it did (Catalogus 1932, p. 20). See also Hondius (2014, pp. 194–195), where it is noted that Bolk was quoted in the so called “neger nummer” (“Negro Number”) of the liberal Amsterdam weekly magazine *De Groene Amsterdammer* (The Green Amsterdammer) by the obstetrician Salomonson (1893–1962), to warn against the “growing Negro cult.”

35 The importance of the population of the Zuiderzee islands extended beyond Dutch borders; even the famous German anthropologist Rudolph Virchow was intrigued by their inhabitants (see: Virchow 1877).

36 A sixth group of 5 bisected Chinese skulls were not included in Bolk’s publication.

37 Bolk received archaeological skulls in the first decade of the twentieth century from Friesland and Zeeland. Even though their provenance was usually no more specific than a general location, that they were archaeological (usually medieval) made them, to Bolk, racially more pure, representing (in Bolk's words) the “original situation’ more than modern Frisian or Zeeland inhabitants (De Rooy 2011, pp. 221–222; Bolk 1908, p. 172).

38 “Meetbare variabelen van den menschelijken schedel en hun onderlinge correlaties in verband met leeftijd en geslacht.”

39 De Froe coordinated excavations on the cemetery of Schokland, one of the former Zuiderzee islands; the skeletal remains were taken to Amsterdam and stored in the anatomical laboratory. De Froe is also known for his attempt to protect the Sephardic Jewish population of Amsterdam from deportation based on Nazi racial theories. He argued they were not Jewish but of the Mediterranean sub-race. Ultimately and unfortunately, this attempt was not successful (e.g. d’Oliveira 2015).

40 Belanda Hitam were a small population of people of African descent living in Indonesia, descendants of Ghanese brought there by the Dutch in the middle of the nineteenth century to serve in the KNIL (The Royal Dutch Indies Army).

41 De Froe and Bijlmer also had a larger series of skulls from (mainly) Dutch origin at their disposal: skulls from Friesland and Zeeland from Bolk’s collection; skulls from Schokland exhumed by De Froe (see note 39); the series of skulls measured for De Froe’s dissertation. But the bottom line is that none of these series, or a smaller sample of them, were included in the series that did include Indonesian, Chinese and “Negro” skulls. They were entities that were grouped and stored completely separately from one another.

42 Modern in the sense that of course the focus on measurements remains up to today, but the focus on race has been set aside, for good and logical reasons.
